# Intestinal microflora and body mass index during the first three years of life: an observational study

**DOI:** 10.1186/1757-4749-3-8

**Published:** 2011-05-23

**Authors:** Carl Vael, Stijn L Verhulst, Vera Nelen, Herman Goossens, Kristine N Desager

**Affiliations:** 1 Department of Microbiology, University of Antwerp, Antwerp, Belgium; 2 Department of Pediatrics, University of Antwerp, Antwerp, Belgium; 3 Department of Health, Provincial Institute for Hygiene, Antwerp, Belgium

## Abstract

**Background:**

Recent research on obesity has demonstrated that the intestinal microflora can have an important influence on host energy balance. The aim of the study was to investigate the relationship between the intestinal microflora and the body mass index in the first 3 years of life.

**Results:**

In a prospective study, a faecal sample from 138 infants was taken at the age of 3, 26 and 52 weeks and cultured on selective media for 6 bacterial genera. Between the age of 1 and 3 years the Body Mass Index Standard Deviation Score (BMI SDS) of these children was determined. The association between the intestinal flora and BMI SDS was assessed for each bacterial genus. A positive correlation was found between the *Bacteroides fragilis *concentration and the BMI SDS at the age of 3 and 26 weeks. The *Staphylococcus *concentration showed a negative correlation with the BMI SDS at the age of 3 and 52 weeks. A low intestinal ratio of *Staphylococcus/Bacteroides fragilis *at the age of 3 weeks, corresponding to a low *Staphylococcus *and a high *Bacteroides fragilis *concentration, was associated with a higher BMI SDS during the first three years of life.

**Conclusion:**

High intestinal *Bacteroides fragilis *and low *Staphylococcus *concentrations in infants between the age of 3 weeks and 1 year were associated with a higher risk of obesity later in life. This study could provide new targets for a better and more effective modulation of the intestinal microflora in infants.

## Background

The prevalence of childhood obesity is reaching epidemic proportions worldwide [1]. The prevention of obesity during early childhood is critical because the high risk of becoming obese adults [2,3]. This increased risk has even been demonstrated in preschool-age children [4,5]. Furthermore, obese children and adolescents develop serious co morbidity, including type 2 diabetes, metabolic syndrome, non-alcoholic fatty liver disease and sleep-disordered breathing [6-8]. While the main accepted cause of obesity is a genetic predisposition coupled with overeating and a lack of physical activity, it is known that other influences may contribute to the development of obesity; environmental pollutants [9], smoking during pregnancy and certain viruses have been implicated in the etiology of obesity [10-12].

Recent research on obesity in humans and mice has demonstrated that the intestinal micro flora can have an important influence on host energy balance. In obese mice and humans the *Firmicutes *were more abundant and the *Bacteroidetes *population was depressed as compared with lean controls [13,14]. Even a single microbial species can have a significant impact on host metabolism. In a study by Backhed et al. [15] germfree mice colonized by *Bacteroides thetaiotaomicron *showed an increased weight gain and fat deposition as compared with germfree mice. The mechanism for this increased adiposity is at least partially the result of a microbial signal that suppresses the fasting-induced adipose factor (FIAF) of the host, resulting in increased production and storage of triglyceride derived fatty acids from the liver. Gut microbiota can further contribute to adiposity by providing the building blocks of triglycerides (short chain fatty acids) through fermentation of plant-derived polysaccharides. Those intestinal species equipped with the most complete set of enzymes for polysaccharide breakdown, are most likely to be involved in obesity.

Epidemiological studies investigating the microbial gut flora in young children from the general population in relation to obesity are scarce. This study evaluates the intestinal flora in infants recruited prospectively from the general population at birth and followed up to the age of 3 years. High intestinal *Bacteroides fragilis *and low *Staphylococcus *concentrations in infants between the age of 3 weeks and 1 year were associated with a higher risk of obesity later in life.

## Methods

Children (n = 138) were recruited prospectively through maternity clinics in urban and rural Flanders between September 2002 and February 2004, as described previously [9]. Selection criteria for enrolment in the study, which is part of the Environmental Health action of the Flemish Ministry of Health and Environment, were vaginal delivery at term and uncomplicated perinatal period. Data on height and weight, demography and risk factors for obesity of the child and the parents were collected by questionnaires at birth and at the ages of 12, 18, 24, 30 and 36 months. Parents filled in height and weight of their children measured at their last visit with their private physician or with the Child & Family Services. BMI was calculated (kg/m^2^) and was converted to BMI SDS (BMI SDS = [BMI child - population mean BMI]/population standard deviation), using Flemish growth curves [16] (Auxology 1.1, Pfizer). The study protocol was approved by the Ethical Committees of the participating institutes. All parents gave written informed consent.

Approximately 2 g of stools was collected into a sterile recipient by the parents at 3, 26 and 52 weeks of age. The sample was sent to the laboratory under anaerobic conditions where it was stored immediately at -70°C until analysis. A total anaerobe count was obtained by quantitative plating of a saline suspension of faeces on Columbia blood agar after 4 days of anaerobic incubation at 37°C. The saline suspension of faeces consisted of 1 g of wet faeces diluted in 10 ml of sterile saline solution, homogenized using a stomacher. Selective media were used to study the bacterial subpopulations [17]. *Bacteroides fragilis *group (subsequently called *Bacteroides fragilis*) was determined quantitatively on *Bacteroides *Bile Esculin agar (BBE) (Becton-Dickinson, Belgium); only black pigmented colonies obtained after 4 days of anaerobic incubation at 37°C were considered [18]. *Bifidobacterium *was determined on mupirocin (100 μg/ml) trypticase-phytone-yeast agar; only colonies smaller than 0.7 mm obtained after 4 days of anaerobic incubation at 37°C were considered [19]. *Lactobacillus *was cultured on LAMVAB medium at 37°C [20]. Green and white colonies obtained after 4 days of anaerobic incubation were considered. *Enterococci *and *Enterobacteriaceae *were cultured after 4 days of incubation at 37°C in ambient air on resp. bile esculin (only black pigmented colonies were counted) and Mc Conckey agar. *Clostridium *counts were obtained after pretreatment of the faecal sample in 80% ethanol for 15 min. and subsequent culture on Columbia blood agar after 4 days of anaerobic incubation at 37°C. *Staphylococcus *was cultured on Mannitol Salt agar (MSA). Both mannitol positive and negative colonies observed after 4 days of incubation at 37°C in ambient air were counted.

Statistical analysis: all data were presented as mean ± standard deviation, median and range or as percentages. Because bacterial counts followed a right-skewed distribution, data were log^10^-transformed. The not normally distributed log transformed counts were compared using non-parametric tests (Friedman rank sum test). Median and range of the bacterial counts were reported as log ^10 ^CFU/g faeces. The detection level was ≥ 3 log CFU/g. The initial design of the study was balanced with questionnaires being distributed every 6 months. However, the dates responding to the last measurements of height and weight were highly variable resulting in an unbalanced design. Furthermore, the analysis was complicated by non-responses to distributed questionnaires. Therefore, we used linear mixed models (a likelihood-based method). This method provides valid results under less restrictive assumptions concerning missing data (missing at random) [21]. The SAS 9.1. (SAS Institute Inc.) program was used for all analyses. The outcome variable was BMI SDS for children between 1 and 3 years of age. Mixed models determine which covariates significantly influence the intercept and slope of BMI SDS for this age range.

The association between the intestinal microflora and BMI SDS was assessed for each bacterial species separately. Second, separate models were built for the cultures at the ages of 3 weeks, 6 months and 12 months. We controlled for possible confounders (maternal BMI, formula or breastfeeding, infant use of antibiotics, parental socio-economic status) and known risk factors of childhood obesity (smoking status of the mother and birth weight SDS). In every mixed model, the interaction of all covariates with time was assesses. Non-significant interactions were deleted from the model.

## Results

The subject characteristics (n = 138) were presented in Table 1. The percentage of missing data for BMI SDS at 12, 18, 24, 30 and 36 months was 5%, 4%, 24%, 38% and 40% respectively.

**Table 1:** Subject characteristics

Variable	Mean ± standard deviation
Number of cases	138
Percentage boys (%)	53.3
Birth Weight (kg)	3.4 ± 0.4
Birth Weight standard deviation score	-0.25 ± 0.92
Birth Length (cm)	50.45 ± 1.78
Birth Length standard deviation score	0.07 ± 0.96
BMI Mother (kg/m^2^)	23.19 ± 3.95
Percentage of households with low income (%)	13.6
Maternal smoking before pregnancy (%)	44.4
Maternal smoking during pregnancy (%)	9.6
Breastfeeding (%) at the age of 3w	73.2
Breastfeeding (%) at the age of 26w	23.9
Breastfeeding (%) at the age of 52w	16.7
Infant use of antibiotics (%) at the age of 3w	1.5
Infant use of antibiotics (%) at the age of 26w	29.9
Infant use of antibiotics (%) at the age of 52w	50.7

The changes in the intestinal microflora during the first year of life were shown in Table 2. The median *Bacteroides fragilis *concentration continued to increase from the age of 3 weeks until the age of 1 year. The median *Staphylococcus *and *Lactobacillus *concentration decreased from week 3 to week 26 and remained stable at week 52. For *Bifidobacterium, Clostridium *and total anaerobes the median concentrations increased from week 3 to week 26 and remained stable at week 52. The median concentration of *Enterobacteriaceae *increased from week 3 to week 26 and decreased again to its starting concentration at the age of 1 year.

**Table 2:** Median counts of faecal microorganisms (log CFU/g) (range) during the first year of life

Bacteria	Week 3	Week 26	Week 52
*Bacteroides fragilis*	0 (0-8.7)	5.8** (0-9.3)	6.2* (0-8.8)
*Enterobacteriaceae*	5.3 (0-9.1)	5.8* (0-8.7)	5.3* (0-8.2)
*Bifidobacterium*	6.1 (0-9.1)	6.8** (0-9.0)	6.5 (0-8.9)
*Lactobacillus*	0 (0-8.6)	0* (0-8.7)	0 (0-8.1)
*Clostridium*	3.8 (0-6.2)	4.3** (0-8.7)	4.5 (0-8.3)
*Staphylococcus*	4.4 (0-8.6)	3.9* (0-8.2)	3.8 (0-8.4)
*Enterococcus*	5.4 (0-9.1)	5.8 (0-8.7)	5.3 (0-8.7)
*Total Anaerobes*	7.7 (0-9.1)	8.1* (3.6-9.6)	7.8 (4.6-9.4)

*p < 0.05 or **p < 0.01 indicates a significant difference in concentration as compared to the previous time period.

The correlation between the intestinal microflora and the BMI SDS during the first three years of life was demonstrated in Table 3. *Bacteroides fragilis *showed a positive correlation with the BMI SDS at the age of 3 and 26 weeks. This correlation demonstrated a negative interaction with the child's age (- 0.02 ± 0.01, p < 0.05), indicating that the effect of *Bacteroides fragilis *on the BMI SDS decreased with the age and disappeared at the age of approximately 2.5 years. *Staphylococcus *showed a negative correlation with the BMI SDS at the age of 3 weeks and 52 weeks. The ratio of *Staphylococcus/Bacteroides fragilis *demonstrated a negative correlation with the BMI SDS only at the age of 3 weeks. At later age (26 and 52 weeks) the negative effect of *Staphylococcus *and the positive effect of *Bacteroides fragilis *on the BMI SDS seemed to cancel each other since no effect of the ratio on the BMI SDS could be shown. A low intestinal ratio of *Staphylococcus/Bacteroides fragilis *at the age of 3 weeks, corresponding to a low *Staphylococcus *and a high *Bacteroides fragilis *concentration, was associated (p = 0.002) with a higher BMI SDS during the first three years of life.

**Table 3:** Correlation between BMI SDS and the intestinal microflora: regression coefficient ± standard error

Bacteria	at age 3 weeks	at age 26 weeks	at age 52 weeks
*Bacteroides fraglis*	0.05 ± 0.02*	0.05 ± 0.02*	- 0.01 ± 0.02
*Staphylococcus*	- 0.04 ± 0.02*	0.04 ± 0.02	- 0.05 ± 0.02*
*Ratio Staph./B. frag*.	- 0.05 ± 0.01**	0.00 ± 0.02	0.01 ± 0.03
*Enterobacteriaceae*	- 0.02 ± 0.02	0.03 ± 0.02	0.02 ± 0.02
*Bifidobacterium*	- 0.02 ± 0.02	- 0.02 ± 0.02	0.00 ± 0.01
*Clostridium*	- 0.02 ± 0.02	- 0.02 ± 0.03	- 0.01 ± 0.02
*Lactobacillus*	0.01 ± 0.01	- 0.01 ± 0.02	- 0.01 ± 0.02
*Enterococcus*	0.01 ± 0.02	0.03 ± 0.02	- 0.02 ± 0.02
*Total Anaerobes*	- 0.03 ± 0.03	0.02 ± 0.04	0.03 ± 0.04

* p < 0.05; ** p < 0.01. P-values were adjusted for confounders (formula and/or breastfeeding, use of antibiotics by the child and socio-economic status) and for known risk factors of childhood obesity (BMI mother, smoking status of the mother and birth weight SDS).

## Discussion

This is a prospective study that demonstrates the influence of the intestinal microflora on the BMI during the first three years of life. High intestinal *Bacteroides fragilis *and low *Staphylococcus *concentrations in infants between the age of 3 weeks and 1 year were associated with a higher BMI SDS in preschool children.

**Figure 1:**
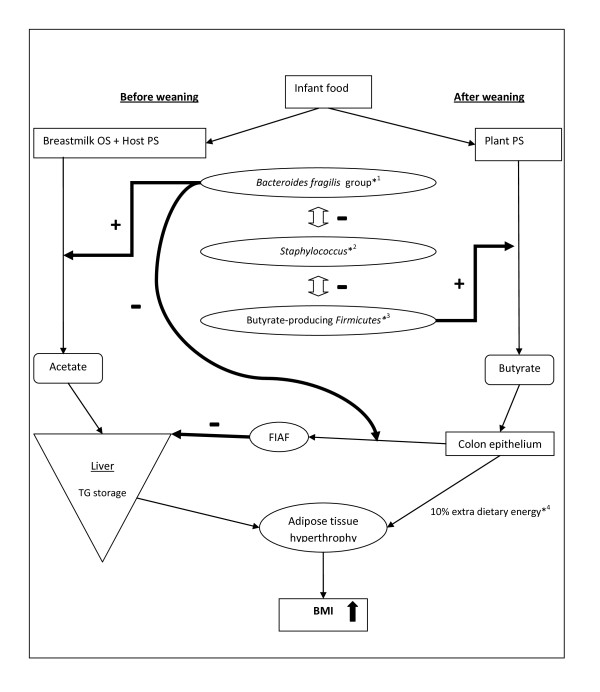
The proposed role of the intestinal microflora in the development of obesity in infants *^1 ^Host-derived polysaccharides can be fermented by *Bacteroides thetaiotaomicron *to predominantly acetate, which is absorbed in the liver leading to de novo triglyceride synthesis [23]. Simultaneously, the FIAF expression by the intestinal epithelium is suppressed by *Bacteroides thetaiotaomicron *resulting in increased production and storage of triglyceride derived fatty acids from the liver [24]. *^2 ^*Staphylococcus *as a marker of delayed acquisition of a complex anaerobic microflora [31]. *^3 ^Subgroup of *Clostridium *cluster XIVa: *Roseburia spp *and *Eubacterium rectale *group [28]. *^4 ^Microbial fermentation may release about 10% of extra dietary energy for host cells [30]. TG: Triglyceride PS: Polysaccharides FIAF: Fasting-induced adipose factor OS: Oligosaccharides Double sided white arrow; up and down: mutual competitive bacterial inhibition

In our study intestinal *Bacteroides fragilis *showed a positive correlation with the BMI SDS, this is in agreement with the results from Bäckhed et al [15] in germfree mice demonstrating increased weight gain and fat deposition after intestinal colonization by *Bacteroides thetaiotaomicron. Bacteroides thetaiotaomicron *is part of the *Bacteroides fragilis *group and is the predominant *Bacteroides *species present in the intestinal microflora of both humans and mice [22]. Plant- or host-derived polysaccharides can be fermented by *Bacteroides thetaiotaomicron *to short chain fatty acids, predominantly acetate, which is absorbed in the liver leading to de novo triglyceride synthesis [23]. Simultaneously, the FIAF expression by the intestinal epithelium of the host is suppressed by *Bacteroides thetaiotaomicron *resulting in increased production and storage of triglyceride derived fatty acids from the liver [24]. The fully sequenced genome of *Bacteroides thetaiotaomicron *incidates that it contains 172 glycosylhydrolases and 163 homologs of starch binding proteins - the largest number in any sequenced prokaryote- enabling it to cleave most of the polysaccharides found in nature [25]. Transcriptome-based analysis of bacterial metabolism indicates that in the suckling mouse gut *Bacteroides thetaiotaomicron *prefers host-derived polysaccharides, as well as oligosaccharides present in mother's milk. After weaning, *Bacteroides thetaiotaomicron *expands its metabolism to exploit plant-derived dietary polysaccharides [26]. This illustrates the superb adaptive survival capability of *Bacteroides thetaiotaomicron *to the breastfeeding-weaning period (Figure 1). In a study on the relation between the human intestinal microflora and weight gain during pregnancy, high *Bacteroides *concentrations were associated with excessive weight gain [27]. These results further emphasize the possible role of the *Bacteroides *group in human energy storage and weight gain.

In other studies on obesity, the *Firmicutes *phyla composition, including *Clostridium *groups (*C. coccoides *group and *C. leptum *group) and *Eubacterium rectale *was compared with the composition of Bacteroidetes phyla (*Bacteroides *group) by sequencing 16S ribosomal RNA genes from stool samples [13,14]. The outcome of these studies cannot be directly compared with our results since we used different methods (culture on selective media) and we studied the intestinal microflora of neonates and infants which is very different from adults. In a study involving a diet to achieve weight loss in obese adults, Duncan et al. [28] did not find a change in the *Firmicutes/Bacteroidetes *proportions, but a significant diet-dependent reduction in the butyrate-producing *Firmicutes *subgroup (*Roseburia spp *and *Eubacterium rectale *group) was demonstrated. These organisms also showed an increased ability to use a variety of starches for growth compared to *Bacteroides thetaiotaomicron *[29]. We hypothesize that during the breastfeeding period the *Bacteroides fragilis *group ferments milk oligosaccharides and host-derived polysaccharides while suppressing FIAF leading to energy storage and weight gain. After weaning, the introduction of solid food will shift the intestinal microflora of the infant gradually towards the adult type microflora with the introduction of the butyrate-producing *Firmicutes *subgroup providing most of the energy requirements of the colon epithelium from plant-derived polysaccharides. This microbial fermentation may release about 10% of extra dietary energy for host cells [30] (figure 1). This shift could explain the decreasing effect of *Bacteroides fragilis *on the BMI SDS with age that we observed in our study.

It remains unknown how *Staphylococcus *can exert a negative effect on BMI SDS. *Staphylococcus *cannot supply extra dietary energy by fermentation of polysaccharides. Moreover, this facultative organism will have to compete with the anaerobic intestinal microflora (*Bacteroides *and butyrate-producing *Firmicutes*). In a Swedish study on the intestinal colonization in infants it was found that *Staphylococcus *(and other facultative organisms) are suppressed as a more complex anaerobic microflora develops because of their inability to compete with these bacteria: the presence of *Staphylococcus *in one year old infants indicated a slow acquisition of a more complex microflora [31]. Intestinal *Staphylococcus *in infants seems to be a marker of delayed acquisition of a complex anaerobic microflora including the polysaccharide fermenting bacteria. This will result in a decreased ability to extract energy from the available food and a lower BMI, as we observed in our study.

Few data on the relationship between intestinal microflora and obesity in children are available, but a recent study by Kalliomaki et al. [32] found lower intestinal *Bifidobacterium *and higher *Staphylococcus aureus *concentrations in obese compared to normal weight children. The design of this study is different since cases (obese) were selected from a cohort and compared to matched controls with normal weight. Different microbiological techniques were also used (FISH and quantitative PCR) and only *Staphylococcus aureus *was detected. Our culture method detected all *Staphylococcus *including *Staphylococcus epidermidis *which is the most predominant intestinal species in breastfed infants [33]. Another reason why the Kalliomaki study cannot be compared to our study is the fact that no faecal sample was taken before the age of 6 months in contrast to our study which included a faecal sample at the age of 3 weeks enabling the detection of early differences in the intestinal microflora before the introduction of solid food. Kalliomaki et al. [32] studied obesity in primary school children at age 7 years which is different from the preschool children during the first three years of life that we studied. However, both preschool age BMI [3,4,34] and primary school age BMI [2,35] are correlated with adult BMI.

Modification of the composition of the intestinal microflora could contribute to prevention and therapy of obesity. Before weaning it would be necessary to restrain the *Bacteroides fragilis *flora in order to obtain a less efficient energy extraction from the available food. After weaning other anaerobic bacteria, probably the butyrate-producing *Firmicutes*, need to be restrained. Further detailed studies of the intestinal microflora in obese children at different ages are required to improve our knowledge of the relation between gut microflora and obesity. This research should provide new methods and tools for a better and thus more effective control of childhood obesity.

## Conclusion

The results of this study suggest that early differences in the composition of the intestinal microflora precede the development of obesity in children. High intestinal *Bacteroides fragilis *and low *Staphylococcus *concentrations in infants between the age of 3 weeks and 1 year are associated with a higher risk of obesity later in life. Modification of the intestinal microflora in infants might represent a new strategy for prevention and therapy of obesity.

## Abbreviations

BBE: *Bacteroides *Bile Esculine; BMI SDS: Body Mass Index Standard Deviation Score; CFU: Colony Forming Unit; FIAF: Fasting-Induced Adipose Factor; MSA: Mannitol Salt Agar

## Authors' contributions

CV was involved in the study design and concept, helped to draft and revise the manuscript and performed the statistical analysis. SV assisted in the statistical analysis and helped to draft and revise the manuscript. VN assisted in the data acquisition and helped revising the manuscript. HG assisted in the data acquisition and helped revising the manuscript. KD was involved in the study design and concept and helped to revise the manuscript. All authors read and approved the final manuscript.

## Competing interests

The authors declare that they have no competing interests.
